# Patterns of mental health services and mood disorder disability pensions: a standard comparison of Finland’s three largest hospital districts

**DOI:** 10.1186/s12888-023-05342-2

**Published:** 2023-11-13

**Authors:** Tino Karolaakso, Reija Autio, Petra Suontausta, Helena Leppänen, Kimmo Suokas, Päivi Rissanen, Martti T. Tuomisto, Sami Pirkola

**Affiliations:** 1 Faculty of Social Sciences (Psychology), Tampere University, Arvo Ylpön katu 34, Tampere, FI- 33520 Finland; 2 Psychiatry, University of Helsinki and Helsinki University Hospital, Helsinki, Finland; 3 Faculty of Social Sciences (Unit of Health Sciences), Tampere University, Tampere, Finland; 4 Faculty of Medicine and Health Technology, Tampere University, Tampere, Finland; 5 Department of Adult Psychiatry, Tampere University Hospital, Tampere, Finland

**Keywords:** Mental health services, Outpatient care, Disability pensions, Mood disorders, Service diversity, DESDE-LTC

## Abstract

**Introduction:**

Mental disorders are one of the most common and disabling health conditions worldwide. There is however no consensus on the best practice of system level mental health services (MHS) provision, in order to prevent e.g. mood disorder disability pensions (DPs). We analyzed the MHS provision between Finland’s three largest hospital districts Helsinki and Uusimaa (HUS), Southwest Finland and Pirkanmaa, with known differences in mood disorder DP risk but presumably equal rates of mood disorder prevalence.

**Methods:**

We used public MHS data analyzed with the standardized DEscription and Evaluation of Services and DirectoriEs for Long Term Care (DESDE-LTC) mapping tool, focusing on all MHS, outpatient care provision, local services without and with gatekeeping, and centralized services. We also collected demographic data based on the European Socio-Demographic Schedule (ESDS). As a novel approach, the Gini-Simpson Diversity Index (GSDI) was calculated for the districts.

**Results:**

Evident differences were observed regarding the districts’ MHS factors. As the hospital district with lower DP risk, HUS was characterized by the highest level of regional socioeconomic prosperity as well as high service richness and diversity. With a nationally average DP risk, Southwest Finland had the highest number of MHS personnel in full-time equivalents (FTE) per 100 000 inhabitants. Pirkanmaa, with a higher DP risk, had overall the lowest service richness and the lowest FTE of the three districts in all MHS, outpatient care and local services with gatekeeping.

**Conclusions:**

Our findings indicate that greater richness and diversity of MHS, especially in outpatient and community-based settings, may serve as indicators of a balanced, high-quality service system that is more effective in preventing mood disorder DP and meeting the different needs of the population. In addition, the need for sufficient resourcing in all MHS and outpatient services is indicated. We suggest using diversity indices to complement the measuring and reporting of regional service variation.

**Supplementary Information:**

The online version contains supplementary material available at 10.1186/s12888-023-05342-2.

## Background

Mental disorders are among the world’s most common and disabling health conditions. Every year over one third of the EU’s population suffers from a mental disorder [1]. In OECD countries, there is evidence that mood disorders are the fastest-growing cause of disability among all mental health disorders, especially among young people [2]. In Finland, mental and behavioral disorders, especially mood disorders, are among the most significant diagnostic groups from which people enter early disability pension (DP) [3].

Current mental health policies have guided MHS system development focusing on outpatient care and community-based care over hospital-focused care systems [4–7]. However, there is no consensus among experts and stakeholders on the best practice of mental health services (MHS) provision on a system level in different contexts (local health area as the meso-level of service organization) [8, 9]. Research literature still lacks information linking MHS characteristics to indicators of service-level effectiveness, for example on the risk of regional DP for mental disorders. In Finland, recent research has identified differences in mood disorder-related disability pensioning risk between the three largest hospital districts: the hospital district of Helsinki and Uusimaa (HUS) had a lower mood disorder DP risk compared to the Finnish national mean, the hospital district of Southwest Finland had a risk level corresponding to the Finnish national average, and the hospital district of Pirkanmaa had a higher risk of DP during 2010–2015 [10]. These risk levels were adjusted based on regional gender, age, and occupational status differences. Previous research has not identified differences in the prevalence of mood disorders between these regions, so the DP differences may partly relate to differences in mental health service systems and local treatment practices [10–12].

In order to study MHS system effects and translate research findings into policy and practice, one must consider the totality of circumstances and context in which the MHS are provided: the local mental health ecosystem. The Mental Health Ecosystems approach is an emerging discipline which takes a whole-systems approach to mental healthcare, enabling the analysis of the complex environment and context of mental health systems, and guiding the translation of this information into policy and practice [9, 13, 14]. In addition to understanding the contextual needs of the population for MHS, a shared language for describing and coding local care delivery context comparable across different regions is required in order to study and compare the features of MHS. Some frequently used and highly sophisticated standardized classification systems for mental health services include the European Service Mapping Schedule (ESMS) and the DEscription and Evaluation of Services and DirectoriEs for Long Term Care (DESDE-LTC; developed from ESMS for the broader assessment of health and social care systems) mapping tools [15–17]. DESDE-LTC has also been called ESMS-R in the original Finnish translation [18–20]. DESDE-LTC provides a standardized taxonomy for describing, classifying, and measuring MHS and their resources. DESDE-LTC has demonstrated high feasibility, consistency, inter-rater reliability and face, content and construct validity [17]. Previously it has been applied to both compare MHS provision between countries and to study the MHS within countries between meso-level regions [21–32]. These studies have found significant variation and critical gaps in MHS provision between both regions and countries. This emphasizes the importance of continuing MHS research to identify and monitor indicators of high-quality services and provide information for experts, policy makers and stakeholders to help organize MHS for the needs of the population and to identify and fill the gaps in MHS provision.

Until 2022, the Finnish municipalities were responsible for organizing public MHS, either by themselves or together via 21 hospital districts [4, 33, 34]. This created significant regional variation and heterogeneity in the Finnish MHS, which unfortunately also relates to the different financial conditions of the municipalities, creating regional inequality and disintegration of MHS provision [20, 34]. At the beginning of 2023, the national reform of healthcare, social welfare and rescue services shifted public MHS provision from the municipalities to the newly founded wellbeing services counties [35]. The wellbeing services counties’ boundaries follow for the most part the boundaries of the old hospital districts. This transfer in organizational responsibility has the potential to support better coordination and integration of regional MHS provision and system development.

This study applies the Mental Health Ecosystems approach in studying the relationship between disability pensioning and MHS provision [13]. The aim of this study was to provide a standard assessment and comparison between these meso-level MHS ecosystems and to analyze their contextual and MHS characteristics in relation to their known differences in mood disorder DP risk (ICD-10 classification F30-39). As the three largest hospital districts in Finland, these study areas provide a representative naturalistic setting in a Nordic welfare country to assess regional MHS with known DP risk differences. We explored the variation in MHS resources and resource allocation as well as service richness and diversity in all MHS, outpatient care, and local community-based and centralized services. As a preliminary hypothesis we hypothesized that lower MHS resources, richness, and diversity, especially in outpatient care and community-based services, could result in critical gaps in service provision which could affect the regional DP risk. Thus, a lower rate of service diversity and resources would be found in higher DP risk regions. To our knowledge there is no prior study assessing MHS ecosystem characteristics on this scale in regard to the regional DP risk differences.

This study is part of the RETIRE – research project, which aims to study the risk factors and sequences of mental health-based disability pensioning and examine the effectiveness of MHS systems in Finland [10, 19, 36–39]. It contributes to the accumulating body of scientific research needed to coordinate and plan MHS and their provision in order to effectively prevent work disability due to mood disorders.

## Methods

### The study catchment area and data collection

**Fig. 1:**
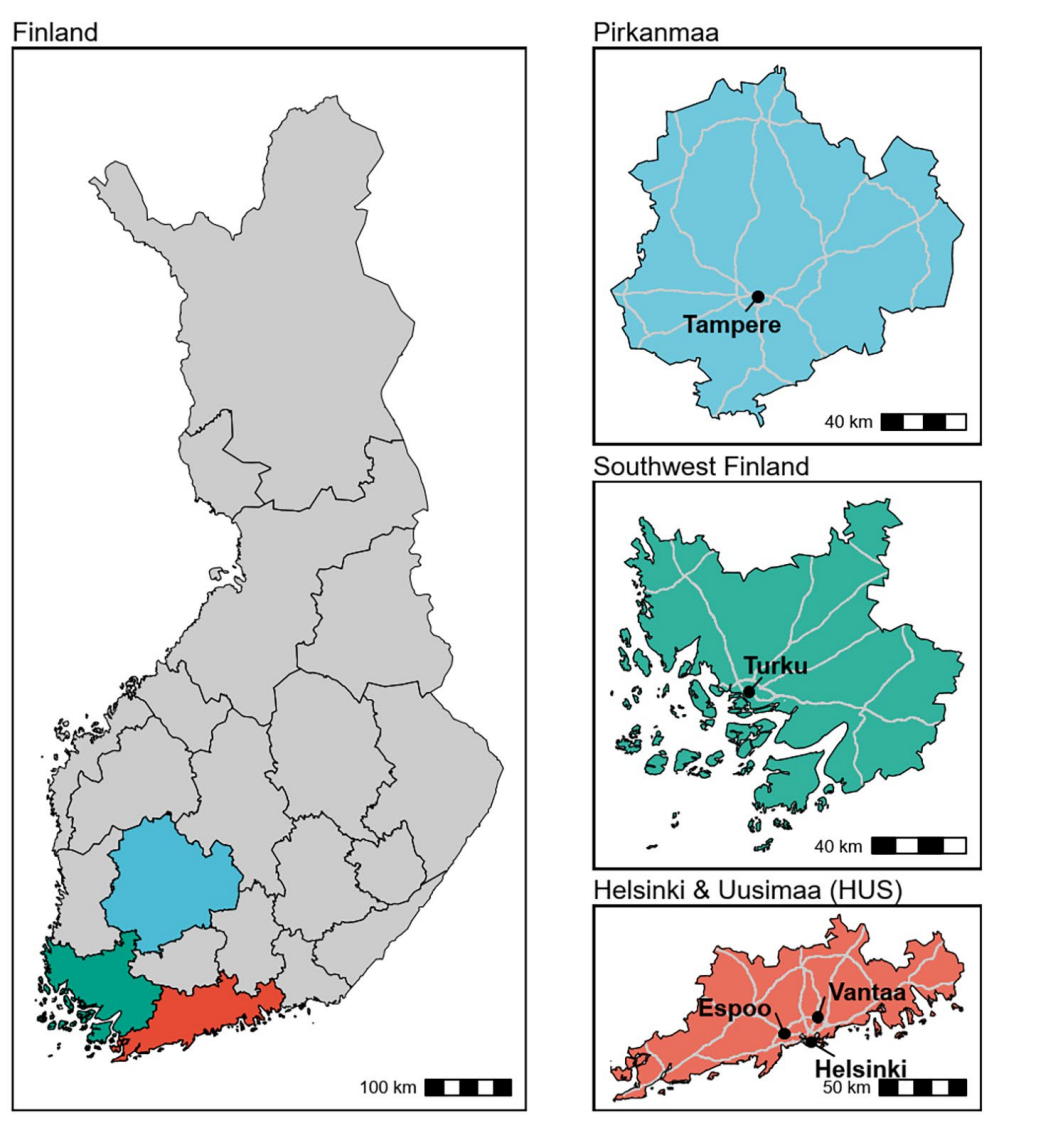
Map of the study’s catchment area

The study’s catchment area consisted of the three most populous hospital districts in the southern, most urban area of Finland, HUS, Southwest Finland and Pirkanmaa (the Tampere Region). The study area is shown in Fig. 1. HUS includes the southern capital area of Finland, along with several other urban cities and semi-urban areas. It is the largest area by population, and produces approximately 40% of the Finnish gross domestic product with the business structure being largely service-oriented. Southwest Finland is situated by the Archipelago Sea, and has a long history of being the most important Finnish trading center from the Middle Ages until the 19th century. Pirkanmaa is one of the fastest-growing regions in the Finnish inland territory. It was mostly a rural region until the 18th century, after which it became one of the main centers of Finnish industrial production. The study areas had a total population of 1 658 457 inhabitants aged 18 to 65 at the end of 2015, and 11 456 first-time mood disorder DP receivers in 2010–2015 as indicated by the data of previous studies [10, 36].

We employed the Mental Health Ecosystems approach to identify and analyze specific sociodemographic and MHS factors relevant in the scope of this study [13]. The hospital districts’ sociodemographic characteristics were collected based on the European Socio-Demographic Schedule (ESDS) for 2015 in order to be congruent with the previously available DP data [40]. ESDS guides which sociodemographic factors from national registers to include into a study. These factors provide the possibility of standardized comparison between areas and countries, they have a studied association with psychiatric disorder rates and service utilisation, and they are similarly collected in several European countries with easy accessibility. The information was gathered from Statistics Finland and the Sotkanet Indicator Bank, an information portal provided by the Finnish Institute for Health and Welfare (THL) that offers essential population health and welfare data [41].

**Fig. 2:**
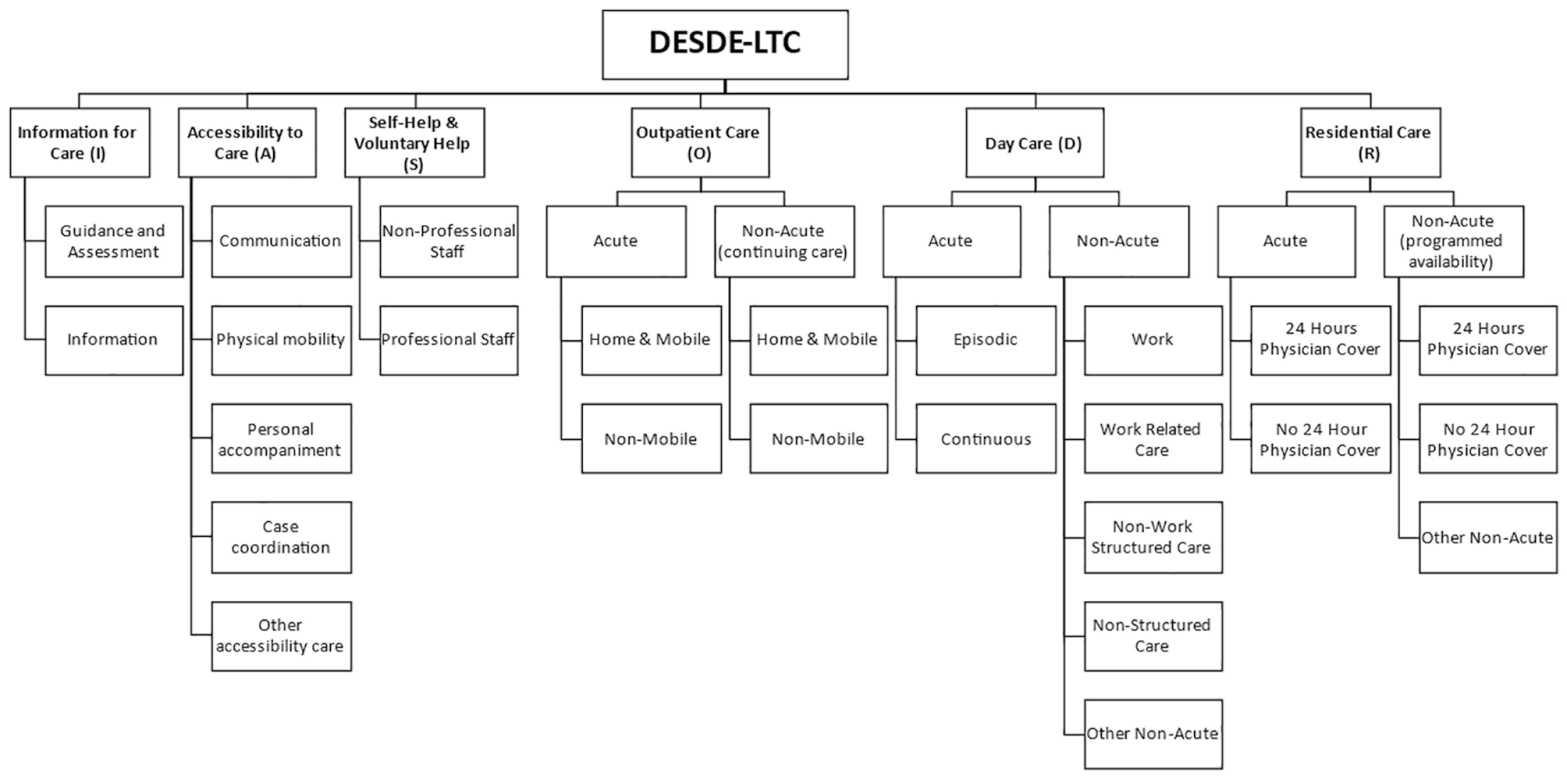
DESDE-LTC classification mapping tree

Using the DESDE-LTC-tool [17], the HUS and Southwest Finland MHS were analyzed during 2012–2013 by the REFINEMENT project (Research on Financing Systems’ Effect on the Quality of Mental Health Care in Europe) [20, 28, 42]. The MHS for Pirkanmaa were analyzed retrospectively for the year 2013. These years correspond with the DP data timespan of 2010–2015 in previous studies [10], as no major alterations were made to these districts’ MHS during these years. The outline and main classes of the DESDE-LTC’s hierarchical taxonomy-based coding tree are presented in Fig. 2. A detailed presentation of the classes is shown in Additional File 1. Although DESDE-LTC has also been called ESMS-R in Finland, we use the internationally more established DESDE-LTC name in this study. The DESDE-LTC data is the most comprehensive available dataset concerning MHS system characteristics and resources in Finland. For more information on DESDE-LTC taxonomy, see for example [17, 28, 30, 43].

### DESDE-LTC processing and analysis

We focused on five different MHS types from the DESDE-LTC -data: (1) all MHS, (2) outpatient care (DESDE-LTC class O), (3) local services without gatekeeping (no referral required), (4) local services with gatekeeping (referral or other specialist assessment required), and (5) centralized services. As prior research has indicated several advantages of local, community-based services, and given the global reforms in MHS that emphasize shifting focus from hospital-centered systems to community-based service systems [5, 7], we used a categorizing variable designed to identify local services with and without gatekeeping and centralized services from the existing DESDE-LTC data [43].

**Fig. 3:**
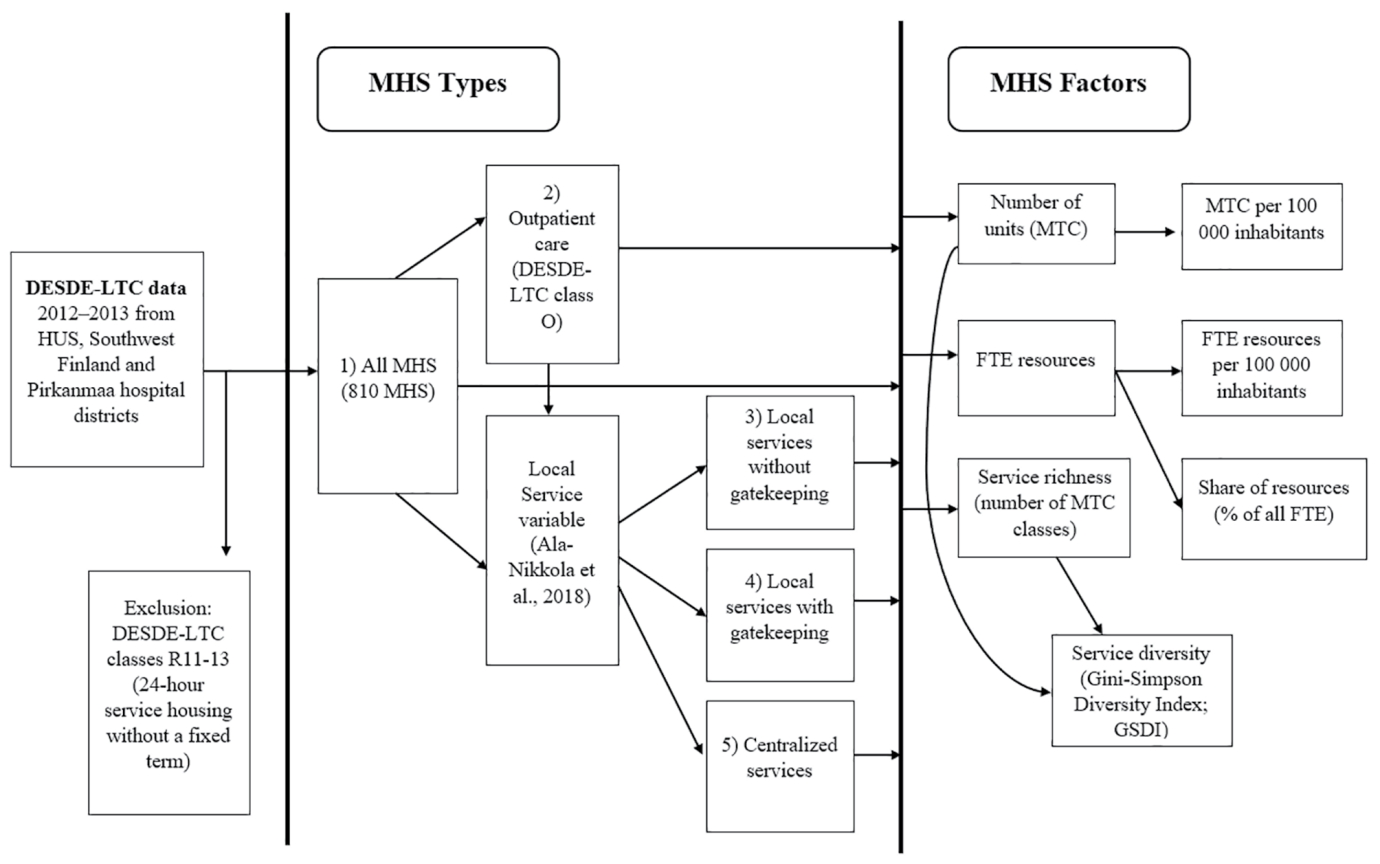
Flowchart of DESDE-LTC data management

For these five different types of MHS, seven DESDE-LTC-service system characteristic factors were calculated for the hospital districts: (1) number of units as main types of care (MTC), (2) MTC per 100 000 inhabitants, (3) service resources as the number of personnel in full-time equivalents (FTE), (4) FTE per 100 000 inhabitants, (5) share of resources, calculated as the personnel FTE percentage of all FTE, (6) service richness as all the different MTC classes in the district, and (7) service diversity as the Gini-Simpson Diversity Index (GSDI) calculated with service richness and the available units for the hospital districts [19, 44, 45]. The flowchart of DESDE-LTC data management is shown in Fig. 3.

Although service richness may seem like a straightforward way to measure service diversity, it only considers the number of reported service classes, rather than the number and evenness of MTC available in the district. Ecological ecosystem service research typically uses the GSDI and similar diversity indices to calculate species or class diversity in a given area [46, 47]. The GSDI, which combines service richness with the number of available MTC in the DESDE-LTC class, enables a different view of MHS diversity by factoring in the evenness of MHS provision in the district’s mental health ecosystem. This approach contrasts with most of the previous studies, which have relied on the number of DESDE-LTC/ESMS-R classes alone (referred to as service richness in this study). A higher GSDI value indicates greater diversity, ranging from 0 to (1) The formulation of GSDI values in this study is shown in Additional File (2) Previously GSDI has been used on a lower spatial scale to calculate a diversity index for municipalities [19]. Here we are using it to measure the service diversity and evenness of larger meso-level districts. To our knowledge this study is the first to assess the applicability of GSDI to the evaluation of MHS diversity in a meso-level regional MHS ecosystem, made possible by the DESDE-LTC taxonomy of MHS.

Services classified as 24-hour supported housing services without a fixed term (DESDE-LTC classes R11-13) are not included in this study, as they are targeted primarily to people already on a DP. With the assistance of a DESDE-LTC specialist, no other services in the DESDE-LTC taxonomy were identified to primarily consist of DP recipients. Furthermore, the FTE information was missing from 111 services, which were excluded from the analysis. The services with missing FTE were predominantly found in the DESDE-LTC main branches of self-help and volunteer care (76.6%), day care (14.4%), outpatient care (4.5%), residential care (3.6%) and information for care (0.9%). When categorized into local services with and without gatekeeping, as well as centralized services, 82% of the services lacking FTE information were local services without gatekeeping, 9.9% were local services with gatekeeping and 8.1% were centralized services. The statistical analyses were performed with R using EpiR packages [48, 49]. The GSDI values were calculated with R version 4.0. [49], RStudio [50] and the R-package *diverse* [51]. Chi-squared-tests and Poisson regression with the number of inhabitants as an exposure were used for obtaining the statistical differences between the hospital districts. The final MHS data in the study area included 810 MHS units with FTE of 5068 and an overall service richness of 63 different MTCs.

## Results

### Study area characteristics

The hospital districts’ demographic characteristics and mood disorder DP differences with the whole of Finland as a comparison, are reported in Table 1. HUS had a population base aged 18 to 65 over three times higher compared to either Southwest Finland or Pirkanmaa, as well as the highest population density. HUS was also characterized by the lowest rate of unemployment, number of households with only one person, population aged 65 and over, and correspondingly the lowest demographic dependency ratio. In addition, HUS had the highest rate of higher education qualifications but also the highest rate of young people aged 17–24 not in education or training.

**Table 1:** Sociodemographic characteristics for the study area hospital districts and for the whole of Finland as a comparison (2015)

	Helsinki and Uusimaa (HUS)	Southwest Finland	Pirkanmaa	Finland	
First time mood disorder F30-39 disability pension (DP) receivers 2010–2015	6 706	2 197	2 553	24 132	
Total population aged 18 to 65	1 045 309	291 768	323 532	3 348 683	
Hospital district differences between mood disorder–related disability pensions DP 2010–2015 by incidence rate ratio (and 95% confidence interval) ^1^	0.84 (0.78–0.90)	1.03 (0.95–1.11)	1.11 (1.03–1.20)	1.00	
Mental health outpatient visits per 1000 persons aged 18 and over	450.8	578.6	346.7	496.2	
Mental health index, not age-standardized	81.40	93.20	112.60	106.4	
Unemployment rate, as % of labour force	11.3%	13.2%	15.3%	13.4%	
Household/dwelling-units with one person, as % of all household/dwelling-units	41.6%	43.5%	42.9%	42.2%	
Population density, population/km^2^	184.7	43.3	36.4	18.1	
Population aged 65 and over as % of total population	16.5%	20.6%	21.7%	20.5%	
Demographic dependency ratio, as the number of people aged under 15 and over 64 per hundred working-age people aged 15–64	50.0	58.9	58.3	58.2	
Higher education qualifications, as % of total population aged 20 and over	36.9%	29.4%	30.7%	30%	
Not in education or training aged 17–24, as % of total population of the same age	10.3%	8.5%	6.9%	8.3%	

^1^ Adjusted based on regional gender, age and occupational status – source: Karolaakso T, Autio R, Näppilä T, Leppänen H, Rissanen P, Tuomisto MT, Karvonen S, Pirkola S (2021) Contextual and mental health service factors in mental disorder-based disability pensioning in Finland – a regional comparison. BMC Health Serv Res 21:1–13 10.1186/s12913-021-07099-4

Southwest Finland and Pirkanmaa were more similar compared to HUS. Pirkanmaa had the highest rate of unemployment as well as population aged 65 and over, and the lowest rate of population density but also the lowest rate of young people aged 17–24 not in education or training. Pirkanmaa had the highest rate of mood disorder DPs, but also interestingly the lowest number of mental health outpatient visits per 1000 persons aged 18 and over. Southwest Finland had the highest rate of households with one person and the lowest rate of higher education qualifications. Demographic dependency ratio was similar between Southwest Finland and Pirkanmaa. Southwest Finland had the highest number of mental health outpatient visits per 1000 persons.

### MHS characteristics

**Fig. 4:**
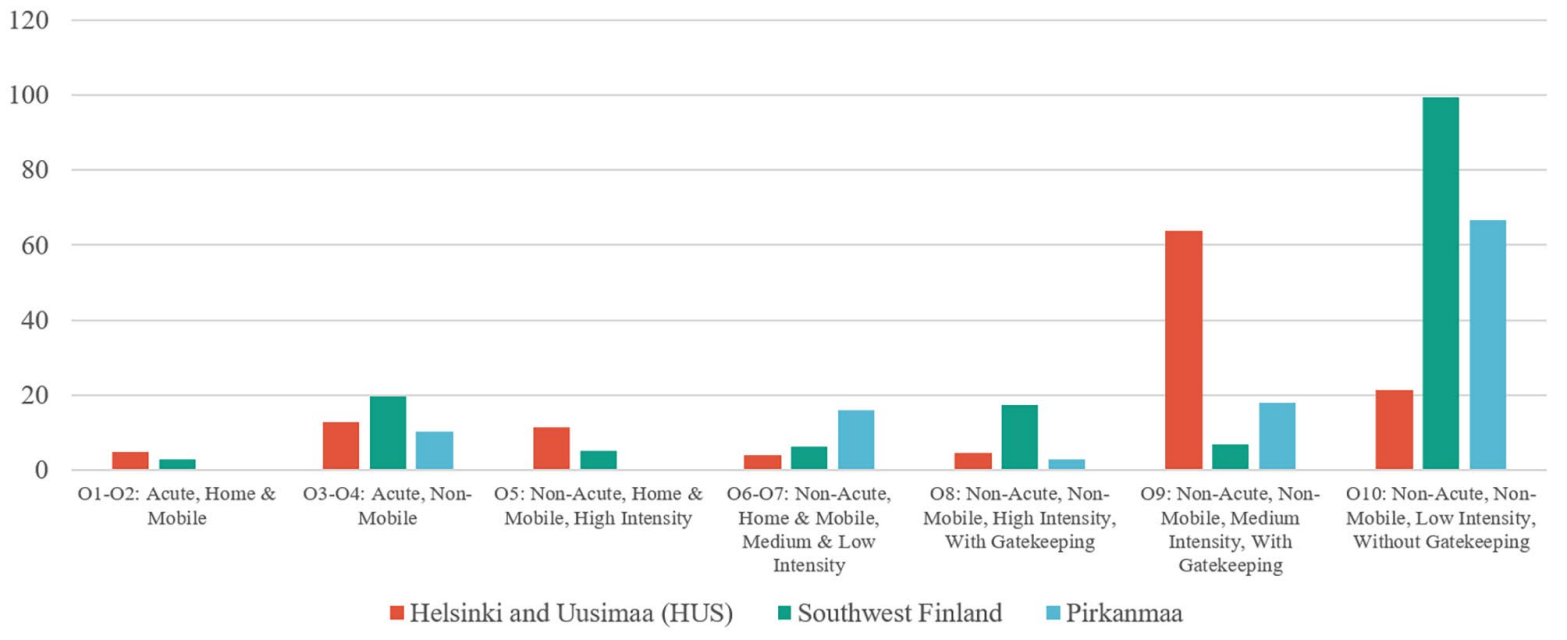
The distribution of resources in outpatient care (O) as the number of personnel in full-time equivalents (FTE) per 100 00 inhabitants

Evident differences between hospital districts were observed regarding the MHS factors (Table 2), as well as variation in the patterns regarding outpatient care allocation (Fig. 4) and DESDE-LTC service class distribution (Additional File 3). In all MHS, HUS (with the largest population and lowest DP risk) had approximately twice as many units but three times the number of FTE than Southwest Finland and Pirkanmaa. HUS had the highest service richness (54 different MTC classes) and the highest service diversity (GSDI of 0.94). HUS also had the highest service diversity in outpatient care (GSDI of 0.80) and in local services without gatekeeping (GSDI of 0.80). HUS was also characterized by a strong emphasis on local services with gatekeeping, comprising 36% of all available FTE and the highest rate of FTE per 100 000 inhabitants (108), but it also had the lowest share of FTE in local services without gatekeeping, which only had 11% of all FTE and the lowest FTE per 100 000 inhabitants (32.8). Overall, HUS had the highest service richness but the lowest rate of MTCs per 100 000 inhabitants in all the MHS types considered. In outpatient care there was a strong emphasis on medium-intensity outpatient clinic services, which mostly had a higher outpatient care visit frequency of at least once in two weeks (class O9: 63.84 FTE per 100 000 inhabitants) and more home delivered, mobile high intensity care (O5) compared to other districts.

**Table 2:** Characteristics of the DESDE-LTC mental health service factors in the three largest Finnish hospital districts. Inhabitants calculated from the population aged 18 to 65

	Helsinki and Uusimaa (HUS)	Southwest Finland	Pirkanmaa	Statistical significance
**All mental health services (MHS)**				
MTC units	416	215	179	
MTC per 100 000 inhabitants	39.8	74.2	55.5	p < 0.001 ^4^
FTE resources ^1^	3107.4	1023.7	936.9	
FTE per 100 000 inhabitants	297	353.4	290.6	p < 0.001 ^4^
Share of all FTE	100%	100%	100%	p = 1 ^5^
Service richness ^2^	54	40	31	p < 0.001 ^4^
Service diversity ^3^	0.94	0.89	0.91	
**Outpatient Care (DESDE-LTC code O)**				
MTC units	145	89	72	
MTC per 100 000 inhabitants	13.9	30.7	22.3	p < 0.001 ^4^
FTE resources ^1^	1286	456.2	366	
FTE per 100 000 inhabitants	122.9	157.5	113.5	p < 0.001 ^4^
Share of all FTE	41%	45%	39%	p = 0.044 ^5^
Service richness ^2^	16	12	10	p = 0.025 ^4^
Service diversity ^3^	0.80	0.65	0.64	
**Local services without gatekeeping**				
MTC units	168	117	83	
MTC per 100 000 inhabitants	16.1	40.4	25.7	p < 0.001 ^4^
FTE resources ^1^	342.7	324.7	244.6	
FTE per 100 000 inhabitants	32.8	112.1	75.9	p < 0.001 ^4^
Share of all FTE	11%	32%	26%	p < 0.001 ^5^
Service richness ^2^	15	13	7	p = 0.01 ^4^
Service diversity ^3^	0.80	0.68	0.67	
**Local services with gatekeeping**				
MTC units	136	48	42	
MTC per 100 000 inhabitants	13	16.6	13	p < 0.001 ^4^
FTE resources ^1^	1130.2	206.3	171	
FTE per 100 000 inhabitants	108	71.2	53	p < 0.001 ^4^
Share of all FTE	36%	20%	18%	p < 0.001 ^5^
Service richness ^2^	19	13	10	p = 0.038 ^4^
Service diversity ^3^	0.85	0.86	0.86	
**Centralized services**				
MTC units	112	50	54	
MTC per 100 000 inhabitants	10.7	17.3	16.7	p < 0.001 ^4^
FTE resources ^1^	1634.4	492.7	521.3	
FTE per 100 000 inhabitants	156.2	170	161.7	p < 0.001 ^4^
Share of all FTE	53%	48%	56%	p = 0.003 ^5^
Service richness ^2^	20	14	14	p = 0.011 ^4^
Service diversity ^3^	0.89	0.90	0.89	

^1^ Resources as the number of personnel in full-time equivalents (FTE)

^2^ Richness as all the different DESDE-LTC-codes for Main Types of Care (MTC) available in the hospital district

^3^ Diversity as the Gini-Simpson Diversity Index calculated with service richness and MTCs

^4^ Statistical differences analyzed with Poisson regression, population used as exposure

^5^ Statistical differences analyzed with Chi-squared test

Southwest Finland, with a DP risk corresponding to the Finnish national average, had the highest number of units (74.2) and FTE (353.4) per 100 000 inhabitants in all MHS. It had the strongest emphasis on outpatient care (45% of all FTE and 157.5 FTE per 100 000 inhabitants) and on local services without gatekeeping (32% of all FTE and 112.1 FTE per 100 000 inhabitants). Overall, Southwest Finland had the highest number of units per 100 000 inhabitants in all MHS types, and the highest rate of FTE in all but local services with gatekeeping (71.2 FTE). Southwest Finland’s psychiatric outpatient clinic services were mainly classified as O10 low intensity services with care visits mainly less often than once every two weeks (99.33 FTE per 100 000 inhabitants). However, Southwest Finland also had the most resourced O8 high intensity outpatient services with care visits mainly at least three times a week (17.33 FTE per 100 000 inhabitants).

Pirkanmaa, with the highest mood disorder DP risk had the lowest number of units and FTE in all but centralized services. It also had the lowest FTE per 100 000 inhabitants in all MHS (290.6), outpatient care (113.5) and local services with gatekeeping (53). Pirkanmaa had the strongest emphasis on centralized services (56%). It had the lowest service richness in all except centralized services where it had the same number of different MTC classes as Southwest Finland (14 different MTC classes). Pirkanmaa’s psychiatric outpatient services were focused on O10 low intensity services, similarly to Southwest Finland, but with approximately two thirds of the FTE per 100 000 inhabitants compared to Southwest Finland (66.68 FTE per 100 000 inhabitants). The data that indicated Pirkanmaa lacked acute mobile services (O1-O2) and mobile high intensity outpatient care (O5) altogether.

## Discussion

In this study we applied the Mental Health Ecosystems approach with comprehensive standardized classification and description of local MHS to provide a standard assessment and comparison between the three largest Finnish hospital districts with known mood disorder DP risk differences but presumably equal rates of mood disorder prevalence [13]. We found major variation in the patterns regarding MHS resourcing and resource allocation, service richness as well as service diversity. These findings point towards the role of organization and structure of regional MHS on the incidence of psychiatric DP for mood disorders. To our knowledge this is the first study to perform a standard comparison between meso-level health care ecosystems examining them related to their previously reported differences in DP risk for mood disorders.

It is important to note that despite national level regulatory legislation and regional steering actions, we found notable dissimilarity in MHS organization in the three largest Finnish hospital districts with rather similar sociocultural contexts, all situated in southernmost, urban Finland. The reasons for this are probably multiple, including historical, socioeconomic and administrative contextual factors [4, 33, 34]. Although the differences in MHS organization may indicate different needs in complex systems, they may also serve as a source of such disparity or inequality that the current, ongoing Finnish service structure reform aims to overcome.

HUS, as the hospital district with the lowest mood disorder DP risk, was characterized by the highest rates of socioeconomic prosperity as well as by high service richness and diversity. This might indicate that the population associated with HUS already has a higher rate of material resources and welfare than on average in Finland, but also has access to diverse services with better possibilities to meet the needs of the population, as well as fewer gaps in MHS provision. Previous research has revealed that 84% of service variation is explained by the size of the catchment area [20], which partly explains HUS’s approximately 1.5- to 2-fold higher service richness compared to Pirkanmaa. Interestingly, HUS only had the highest FTE per 100 000 inhabitants in local services with gatekeeping, where most of its outpatient care resources were allocated to O9 medium intensity polyclinic services. This contrasted with Southwest Finland and Pirkanmaa, where most of the outpatient resources were classified as O10 low intensity services, implying an interval longer than two weeks between most of the care visits for patients. It is important to note that because these MTCs are classified corresponding to the true interval between most of the provided outpatient care visits, this might point to the importance of the services being able to respond to treatment needs with sufficient appointment frequency regardless of whether the services have gatekeeping or not. Interestingly, HUS also had the lowest MTC per 100 000 inhabitants in all MHS types, but the high FTE per 100 000 inhabitants indicates that these MTC are larger on average FTE-wise compared to Southwest Finland and Pirkanmaa.

Regardless of these considerations, higher FTE per 100 000 inhabitants appear not necessarily to indicate a lower regional DP risk, as Southwest Finland had a higher FTE per 100 000 inhabitants in most of the studied MHS types compared to HUS. The number of FTE and outpatient visits per 1000 inhabitants did show a similar pattern, with Southwest Finland having the highest and Pirkanmaa the lowest numbers of both parameters. Nevertheless, comparing Southwest Finland and Pirkanmaa, two hospital districts with almost equal population bases, Southwest Finland clearly had a higher overall FTE as well as higher service richness in all studied MHS types except centralized services. Pirkanmaa also had the lowest FTE per 100 000 inhabitants of the three districts in all MHS, outpatient care and local services with gatekeeping. In addition, prior research has identified Pirkanmaa as having one of the lowest rates of outpatient visits per Finnish population rate [10]. These observations suggest that possible factors connected to Pirkanmaa’s higher DP risk might include the regional MHS being under-resourced and therefore unable to produce an adequate level of outpatient care to meet the needs of the population. Furthermore, the lower service richness pointed to some vital treatment gaps in service provision, with a lack of acute mobile services (O1-O2) and mobile high intensity outpatient care (O5) and most of the resources allocated to centralized services compared to the MHS systems of HUS or Southwest Finland. Previously, an expert committee has voiced these same concerns and the need for MHS system development in Pirkanmaa [52].

In this study we also applied GSDI as a service diversity indicator in meso-level districts. GSDI implied a significantly higher service diversity for HUS in outpatient care and local services without gatekeeping, and a slightly higher diversity in all MHS. Because the GSDI considers service richness but gives more weight to service evenness of MTC units, this implies a more even distribution of MTCs over more MHS classes in HUS compared to Southwest Finland or Pirkanmaa in all MHS, outpatient care and local services without gatekeeping. However, in local services with gatekeeping and centralized services, the three hospital districts had approximately the same GSDI, but HUS had a distinctly higher service richness. These observations imply that GSDI can work as an important complementary indicator for service diversity and evenness, but that service richness is also needed to understand the overall variation in regional MHS provision.

### Strengths, limitations, and future research

One of strengths of this study was the use of DESDE-LTC tool to analyze MHS provision. DESDE-LTC provides an internationally approved set of systems indicators, classification of services and terminology [15, 17, 30]. DESDE-LTC data collection and analysis included obtaining the MHS information through interviews with local organization supervisors, which has been indicated to have higher validity than using only the official services listings [27, 30].

Our study setting includes some limitations. One central limitation was that our MHS data did not include information concerning the pathways of care or connections between the different mental health service points, or on the treatment culture or customs of the local MHS. Without this information, some aspects of the complex dynamics in these MHS ecosystems are missing. In future research, it would be important to collect information on the dynamics and care pathways between different regional MHS, as well as treatment contents (for example whether evidence-based treatment models are habitually used) and treatment cultures, which could be reported with the other MHS ecosystem information.

It is also important to note that some features of the MTC might be outside the scope of the DESDE-LTC classification tool and might not be included in the analysis. The regional MHS data for the districts only consists of public services, and information on private services, Finnish occupational health care and rehabilitative psychotherapy imbursed by the Social Insurance Institution of Finland was not collected. Earlier research suggests, however, that compared to these other services, the public MHS plays an essential role in addressing and preventing mental disorders and disabilities among the majority of the population [53, 54]. Furthermore, a broad range of other services, including local social, education, housing, justice and employment services as well as employment opportunities in different work and industrial sectors presumably play a role in regional DP outcomes in the case of people on the verge of DP. Considering these services and differences would be an important topic for further research.

Regarding the use of GSDI, the index values are comparable only if the catchment areas are on the same spatial scale [47]. This means that for example district-level and municipality-level GSDI values are not comparable. This must be kept in mind when comparing different study results with different scales. Nevertheless, GSDI and other diversity indices can be an important complementary part of future MHS research when comparing the service provision and diversity of different MHS ecosystems on the same spatial scale.

## Conclusions

Our findings highlight the potential role and importance of the organization and provision of MHS in affecting the regional mood disorder-based DP risk. Greater richness and diversity of MHS, especially in outpatient, and community-based settings, may serve as an indicator of a well-developed and -balanced, high-quality service system that is more effective in preventing mood disorder DP and meeting the different needs of the population. Our findings also point to the role of sufficient resourcing in all MHS and outpatient services, so that essential outpatient clinics can provide psychosocial treatment answering to individual and populational needs.

To understand MHS ecosystems, the use of several different demographic and MHS indicators is essential. We present the possibility of using diversity indices to complement the measuring and reporting of regional service variation in addition to service richness. The diversity and richness of MHS provision should be accounted for in the development of MHS by experts and stakeholders to offer services matching population needs.

In Finland, the ongoing health and social service structure reform and the work to implement The Finnish Mental Health Strategy 2020–2030 create a productive basis to promote broad, effective, and accessible MHS that meet people’s needs [6, 55, 56]. However, despite the prior national-level regulatory legislation and regional steering, notable differences in MHS organization have arisen even between the three largest hospital districts in Finland, acting as a potential source of significant regional inequality in mental health outcomes. In the ongoing work, national cooperation and joint service development is of paramount importance in order to avoid past mistakes creating fragmented services and to ensure equal, high-quality services for all.

### Electronic supplementary material

Below is the link to the electronic supplementary material.

Supplementary Material 1

Supplementary Material 2

Supplementary Material 3

## Data Availability

DESDE-LTC data regarding the hospital districts is available from the Finnish Institute for Health and Welfare. The demographic factors are publicly available from the Sotkanet Indicator Bank, an information portal provided by THL. The aggregated data used and analyzed during the current study is available in Additional File 3. The original DESDE-LTC dataset used is available from the corresponding author on reasonable request.

## Abbreviations

DP: Disability pension
DESDE-LTC: DEscription and Evaluation of Services and DirectoriEs for Long Term Care
ESDS: The European Socio-Demographic Schedule
ESMS-R: European Service Mapping Schedule-Revised
FTE: Full-time Equivalents
GSDI: Gini-Simpson Diversity Index
HUS: Helsinki and Uusimaa Hospital District
MHS: Mental health services
MTC: Main Types of Care
REFINEMENT: Research on Financing Systems’ Effect on the Quality of Mental Health Care in Europe
THL: Finnish Institute for Health and Welfare
