# Supplementary material for: Patterns of mental health services and mood disorder disability pensions: a standard comparison of Finland’s three largest hospital districts
Source: BMC Psychiatry. 2023 Nov 13;23:828. doi: 10.1186/s12888-023-05342-2 (PMC10644417; doi:10.1186/s12888-023-05342-2)
Supplement: Supplementary file 2 — Supplementary Material 2 [file 12888_2023_5342_MOESM2_ESM.pdf]

Additional File 2. Calculating the Gini-Simpson Diversity Index (GSDI).

The Simpson's index *D* (Simpson, 1949) means the probability that any two individuals randomly picked from an infinitely large population will belong to the same species or class. Commonly it is applied as (1 – *D*), which is known as the Gini-Simpson Diversity Index (GSDI). The GSDI thus measures the probability that two randomly picked individuals belong to different species. GSDI is also known as the Gibbs-Martin index or the Blau index in psychology sociology, and management studies.

The GSDI values were calculated using R version 4.0.1 (R Core Team, 2020) and the R-package *diverse* (Guevara, M. R., Hartmann, D., Mendoza, 2016).

For a given area, and in this study for one hospital district, the GSDI was calculated as follows:

$$1 - \sum_{i} (p_i^2)$$

where:

- p_i is the "species" (DESDE-LTC class) proportion, $p_i = x_i \,/\, N$.
- N is the total number of individuals (in this case the available MTCs) in the community (hospital district)

By combining and weighing service richness with the number of available units in the DESDE-LTC class, the GSDI defines an index of 0 to 1 for the MHS diversity of the hospital districts. A GSDI close to 1 signifies that there are several DESDE-LTC classes in the hospital district, and that the available MHS unit proportion of DESDE-LTC classes is even. A low GSDI indicates that the hospital district's MHS is not diverse. For example, if there would be only one class of MHS according to the DESDE-LTC taxonomy in the hospital district, the GSDI is 0.

Example: HUS has 145 outpatient care (O) MTCs available to its population. These 145 MTCs belong to 16 different DESDE-LTC classes, so the service richness of HUS's outpatient care is 16. Of these services, for example O1.1 has one unit, O2.1 has three units, O3.1 has two units, O4.1 has ten units et cetera.

Thus, following the above the formula, the GSDI for HUS's outpatient care is calculated:

$$1 - \left((1/145)^2 + (3/145)^2 + (2/145)^2 + (10/145)^2 + (3/145)^2 + (6/145)^2 + (2/145)^2 + (13/145)^2 + (1/145)^2 + (4/145)^2 + (1/145)^2 + (2/145)^2 + (8/145)^2 + (44/145)^2 + (44/145)^2 + (1/145)^2\right)$$

= GSDI is approximately 0.7959096
