# Supplementary material for: Patterns of mental health services and mood disorder disability pensions: a standard comparison of Finland’s three largest hospital districts
Source: BMC Psychiatry. 2023 Nov 13;23:828. doi: 10.1186/s12888-023-05342-2 (PMC10644417; doi:10.1186/s12888-023-05342-2)
Supplement: Supplementary file 3 — Supplementary Material 3 [file 12888_2023_5342_MOESM3_ESM.pdf]

Additional File 3. The distribution of mental health services and resources in the three largest Finnish Hospital districts according to the DESDE-LTC taxonomy. Data collected in 2012-2013.

Local Service variable classification = A: Local services without gatekeeping; B: Local services with gatekeeping; C: Centralized services

		**MTC units**			**FTE resources**			**FTE per 100 000 inhabitants**		
	Local Service - variable	Helsinki and Uusimaa (HUS)	Southwest Finland	Pirkanmaa	Helsinki and Uusimaa (HUS)	Southwest Finland	Pirkanmaa	Helsinki and Uusimaa (HUS)	Southwest Finland	Pirkanmaa
O1.1	B	1	-	-	12	-	-	1.15	-	-
O1.2	A	-	1	-	-	8	-	-	2.76	-
O2.1	B	3	-	-	39	-	-	3.73	-	-
O3.1	C	2	-	2	49.8	-	9.1	4.76	-	2.82
O4.1	B	10	5	2	69.7	50.8	23.7	6.66	17.54	7.35
O4.2	B	3	3	-	14.5	6.5	-	1.39	2.24	-
O5.1.1	B	6	-	-	33.2	-	-	3.17	-	-
O5.1.2	B	2	1	-	18	1.5	-	1.72	0.52	-
O5.2.1	B	13	4	-	59.7	13.5	-	5.71	4.66	-
O5.2.2	B	1	-	-	8.5	-	-	0.81	-	-
O6.1	B	4	-	6	24	-	14.1	2.29	-	4.37
O6.2	B	-	1	6	-	1.5	20.4	-	0.52	6.33
O7.1	B	1	4	1	3	9.5	17	0.29	3.28	5.27
O7.2	B	2	2	-	14.9	7	-	1.42	2.42	-
O8.1	B	8	14	1	47.7	50.2	9	4.56	17.33	2.79
O9.1	B	44	3	9	668	20	56.7	63.84	6.90	17.58
O9.2	B	-	-	1	-	-	1	-	-	0.31
O10.1	A	44	50	41	223	286.7	201	21.31	98.98	62.34
O10.2	A	1	1	2	1	1	14	0.10	0.35	4.34

R2	C	20	9	8	427.9	184.1	177.4	40.89	63.56	55.02
R3.1.1	C	4	2	3	77	32.9	43.3	7.36	11.36	13.43
R3.1.2	C	-	3	-	-	22	-	-	7.60	-
R4	C	10	5	1	273.1	98.4	20.5	26.10	33.97	6.36
R5	C	1	-	-	3.6	-	-	0.34	-	-
R6	C	22	3	7	404.3	49.3	133.2	38.64	17.02	41.31
R8	C	1	-	-	22	-	-	2.10	-	-
R8.1	C	7	-	2	87.1	-	27.3	8.32	-	8.47
R8.2	C	4	5	3	82.5	35.6	19.7	7.88	12.29	6.11
R9	C	2	-	-	23.2	-	-	2.22	-	-
R9.1	C	1	-	-	3.6	-	-	0.34	-	-
R9.2	C	3	3	9	30	9	22.5	2.87	3.11	6.98
R14	C	4	-	-	14	-	-	1.34	-	-
D0.1	C	-	1	-	-	7	-	-	2.42	-
D1.2	C	9	1	6	53	4	30.8	5.07	1.38	9.55
D2.2	C	2	1	-	2	1	-	0.19	0.35	-
D3	C	-	-	1	-	-	1	-	-	0.31
D3.1	C	3	7	2	11	19	23.3	1.05	6.56	7.23
D3.2	C	12	5	3	48.4	16	7	4.63	5.52	2.17
D4.1	B	16	7	7	53.1	33.8	12.1	5.07	11.67	3.75
D4.2	C	1	-	-	4	-	-	0.38	-	-
D4.3	B	8	1	-	20.3	4	-	1.94	1.38	-
D4.4	A	1	-	4	2	-	8	0.19	-	2.48
D5	A	10	3	-	47.2	2	-	4.51	0.69	-
D6.1	B	-	1	-	-	3	-	-	1.04	-
D7.1	C	2	4	1	8	12.5	-	0.76	4.32	-
D7.2	C	2	1	6	10	2	6.2	0.96	0.69	1.92
D8.1	B	4	2	6	20.5	5	13	1.96	1.73	4.03
D8.3	B	8	-	2	24.1	-	4	2.30	-	1.24
D8.4	A	-	1	-	-	4	-	-	1.38	-
D9	A	12	7	15	21.3	9	17.6	2.04	3.11	5.46

S1.1	A	33	42	-	NA	NA	-	NA	NA	-
S1.2	A	47	-	19	NA	-	NA	NA	-	NA
S1.3	A	3	1	1	NA	NA	NA	NA	NA	NA
S1.4	A	3	4	-	NA	NA	-	NA	NA	-
S2.1	A	1	1	-	NA	NA	-	NA	NA	-
S2.2	B	1	-	-	NA	-	-	NA	-	-
A4	A	5	-	-	23.9	-	-	2.28	-	-
I1.1	A	4	3	1	14.1	7	4	1.35	2.42	1.24
I1.2	B	1	-	-	NA	-	-	NA	-	-
I1.3	A	2	1	-	2	2	-	0.19	0.69	-
I2.1	A	1	-	-	3.2	-	-	0.31	-	-
I2.1.1	A	-	2	-	-	5	-	-	1.73	-
I2.2	A	1	-	-	5	-	-	0.48	-	-
